# The impact of pre-existed and SERM-induced non-alcoholic fatty liver disease on breast cancer survival: a meta-analysis

**DOI:** 10.7150/jca.44872

**Published:** 2020-05-18

**Authors:** Changjun Wang, Yidong Zhou, Wei Huang, Ziyuan Chen, Hanjiang Zhu, Feng Mao, Yan Lin, Xiaohui Zhang, Songjie Shen, Ying Zhong, Xin Huang, Chang Chen, Qiang Sun

**Affiliations:** 1 Department of Breast Surgery, Peking Union Medical College Hospital, Beijing, China; 2 Department of Dermatology, 90 Medical Center Way, Surge 110, University of California, San Francisco, CA 94143-0989, United States

**Keywords:** breast cancer, non-alcoholic fatty liver disease, endocrine therapy, selective estrogen receptor modulator, aromatase inhibitor, liver metastasis

## Abstract

**Background**: Non-alcoholic fatty liver disease (NAFLD) is a common disorder and a frequent side effect of endocrine therapy (ET) for breast cancer treatment. This was the first meta-analysis to investigate the impact of NAFLD on breast cancer survival.

**Material and Methods**: We searched Pubmed, Embase and Cochrane Central Register of Controlled Trials database for relevant studies that investigated the correlation between NAFLD and breast cancer survival. Fixed- and random-effect meta-analyses were conducted according to the heterogeneity of enrolled studies. Subgroup analyses were performed based on whether NAFLD was induced by ET administration

**Results**: Eight cohorts from six studies including 3684 breast cancer patients were enrolled. NAFLD was significantly associated with advanced age (*p* < 0.001), obesity (*p* < 0.001), lymph node metastases (*p* = 0.003) and hormone receptor positivity (p < 0.001). NAFLD had no significant impact on disease free survival (DFS) [hazard ratio (HR) 1.07, 95% confidence interval (CI) = 0.64-1.77, *p* = 0.81] and overall survival (OS) (HR 1.29, 95% CI = 0.68-2.44, *p* = 0.44). In subgroup analyses, ET-associated NAFLD showed no significant impact on DFS and OS. Nonetheless, non-ET-associated NAFLD had a strong prognostic correlation with poor OS (HR 1.92, 95% CI = 1.09-3.41, *p* = 0.02).

**Conclusion**: NAFLD had no significant impact on breast cancer survival. However, non-ET-associated NAFLD implied increasing death risk. Future large-scale studies are warranted to further elucidate the correlation between NAFLD and breast cancer prognosis.

## Introduction

Non-alcoholic fatty liver disease (NAFLD) is one of the most common chronic liver disorders. In Asia, its incidence reaches up to 25%^1^. NAFLD is defined as a condition characterized by excessive fat accumulation (steatosis) in the absence of excessive alcohol consumption (a typical threshold being <30g per day for men and <20g per day for women). It has a wide spectrum of clinical manifestation including simple steatosis, fatty infiltration plus inflammation, and nonalcoholic steatohepatitis (NASH)^2^,^3^. Up to 20% of patients with NAFLD progresses to NASH, which is defined as steatosis coexisting with liver-cell injury and inflammation (steatohepatitis), progressing to fibrosis and ultimately cirrhosis which is the most advanced form of NAFLD^4^. Central adiposity, insulin resistance and weight gain are major risk factors for NAFLD, and genetic predisposition is also another possible explanation for NAFLD susceptibility in non-obese population^1^.

Breast cancer is the most prevalent female cancer worldwide and one of the principal causes for women death^5^. Endocrine therapy (ET), including mainly selective-estrogen receptor modulator (SERM) and aromatase inhibitor (AI) played an important role in adjuvant systemic therapy for hormone receptor positive breast cancer. However, long-term ET could lead to severe adverse effects, such as increasing risk of thromboembolism, endometrial cancer, vaginal bleeding and NAFLD^6^-^8^. ET-associated NAFLD had been widely studied recently and its presence could potentially compromise breast cancer survival. Study by Pan *et al.* suggested that tamoxifen was associated with increased risk of newly developed fatty liver and worsening previous existed NAFLD, even retarding fatty liver recovery 9.

Up to now, the correlation between NAFLD and breast cancer prognosis remained contentious. Several studies proved patients with NAFLD had worse prognosis^10^,^11^, while study by Wu *et al.* suggested NAFLD served as a protective factor towards breast cancer progression^12^. Additionally, given that one of the major ET adverse effect was NAFLD, it remained undetermined that whether NAFLD induced by ET had similar prognostic value as common NAFLD. Two studies by Zheng *et al*. and Yan *et al*. both focused on the impact of ET-associated NAFLD on breast cancer. The former suggested that among the ET users, ET-associated NAFLD patients had better disease free survival (DFS) and overall survival (OS) than non-NAFLD patients, while the latter drew the contradictory conclusion^10^,^13^. Hence, our study reviewed the available publications on Pubmed, Embase, Cochrane Central Register of Controlled Trials (CENTRAL) database and conducted the present meta-analysis to explore the influence of NAFLD on breast cancer survival.

## Materials and Methods

### Data Sources and Search Strategy

The databases including: Pubmed, Embase and CENTRAL database were searched for relevant papers from inception to August, 2019. The search performed using Medical Subject Headings/EMTREE and free-text terms and combining into <terms related to breast cancer > AND <terms related to Non-alcoholic Fatty Liver Disease>, and no language limitation was applied. Unduplicated references of included studies were manually screened by two reviewers (Changjun Wang and Wei Huang). Detailed search strategy please see Supplementary material 01 - Search strategy.

### Selection Criteria

Two reviewers (C.J. Wang and W. Huang) reviewed the title/abstract and then the full-text articles and selected articles independently. Eligible studies must meet the following inclusion criteria: case-control, cross-sectional or cohort studies; studies that evaluated the risk of metastasis of breast cancer among the patients with NAFLD compared with who without NAFLD; studies reported the hazard ratio (HR) of DFS, OS with 95% confidence interval (CI). Potentially relevant articles were reviewed in full text by the same two reviewers. Disagreement was resolved by consensus (C.J. Wang, W.Huang, and Q. Sun).

### Data Extraction

Cohort level characteristics (title of the study; publication year; country; design of the study; number of patients; clinicopathological characteristics of patients; assessment of NAFLD, etc.) were extracted into a structured data collection form for statistical analysis. “ET-associated NAFLD” was defined as patients who did not have pre-treatment NAFLD and developed NAFLD after the initiation of endocrine therapy. “Non-ET-associated NAFLD” was defined as pre-existing (pre-treatment) NAFLD regardless of hormone receptor status. The HR, 95% CI and *p* value were directly extracted from the text and tables of eligible articles. For the study that did not provided HR but Kaplan-Meir curves of DFS or OS, Engauge software was used to estimate the HR and 95% CI from the obtained data^14^.

### Statistical Analysis

Data analysis was performed using the Review Manager (RevMan) [Computer program]. Version 5.3. Copenhagen (The Nordic Cochrane Centre, The Cochrane Collaboration, 2014) and Stata/SE 14.1 for Mac (64-bit Intel) (Revision 01 Dec 2015 Copyright 1985-2015 Stata Corp LP. HR and 95% CI of DFS and OS, were taken directly from the article for meta-analysis. Random effects models were used for meta-analysis when a significant heterogeneity existed between included studies (I-square>50%). Fixed effects models were used when there was no significant heterogeneity (I-square<50%). Data on clinicopathological characteristics between subgroups were tested with Pearson Chi-square test or Fisher exact test.

Heterogeneity among included studies was assessed by the I-square statistic which shows the total variation across studies that is not a result of chance, and I-square>50% indicated that a significant heterogeneity existed. Funnel plot and Begg's test were used to assess the presence of publication bias.

## Results

**Figure 1:**
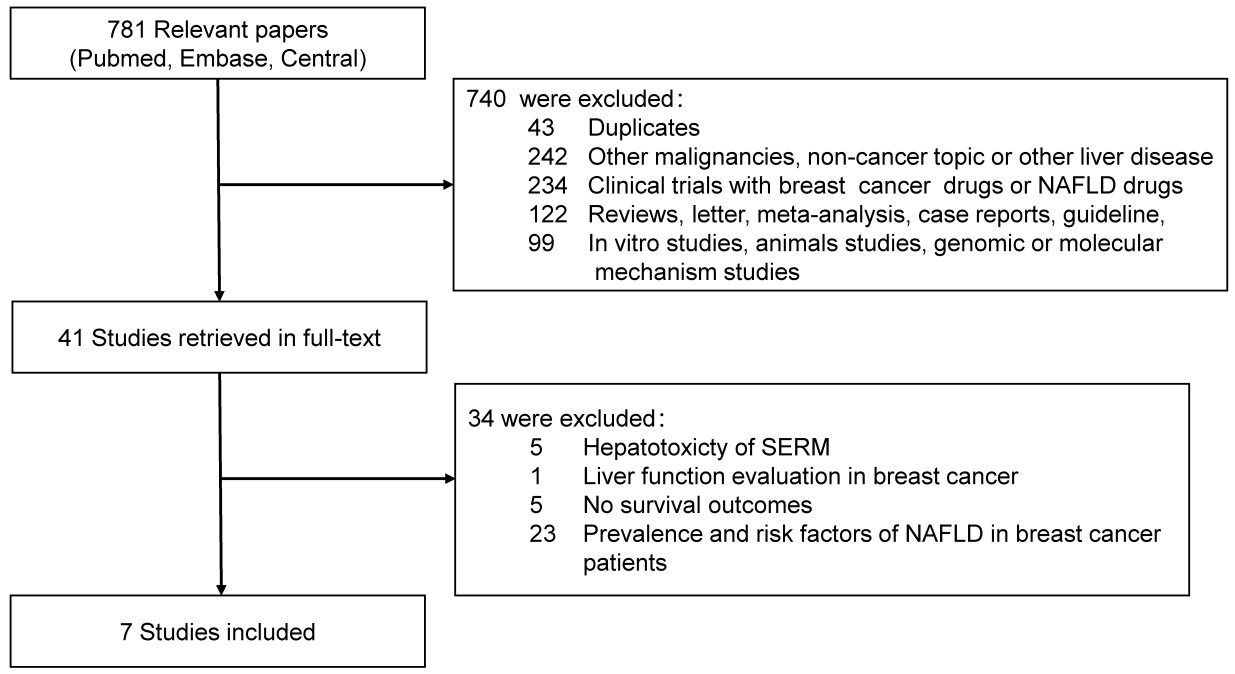
Flowchart of articles reviewed and included in meta-analysis

Totally, 781 relevant studies were extracted from the databases, 41 full-text articles were retrieved for detailed evaluation. Ultimately seven studies with eight cohorts, 3684 patients were enrolled in this meta-analysis 10-13,15-17. Flowchart of literature selection was shown in Fig. 1. All the studies were retrospective cohort studies. Study by Yang *et al.* contained two cohorts and reported DFS separately and OS combined. Three studies selected only hormone receptor positive patients treated with ET. Most of the enrolled patients underwent abdominal ultrasonography for NAFLD diagnosis and assessment, and only study by Duran et al. and parts of participants in study by Yang et al. were assessed by contrast-enhanced or non-enhanced computed tomography. The characteristics of enrolled cohorts were summarized in Table 1.

### Clinicopathological characteristics between NAFLD group and non-NAFLD group

There was no statistically significant difference in tumor size, menopause status, radiotherapy or chemotherapy status between NAFLD group and non-NAFLD group. While NAFLD was prone to affect elderly patients ( < 50 years versus age > 50 years, 24.7% versus 32.4%, *p* < 0.001) with obesity [Body mass index(BMI) < 25 kg/m^2^ versus BMI > 25kg/m^2^, 25.4% versus 46.7%, *p* < 0.001], lymph node metastasis (lymph node metastasis versus no lymph node metastasis, 40.9% versus 36.6%,* p* = 0.003) and hormone receptor positivity (positive versus negative, 34.9% versus 14.1%, *p* < 0.001). The correlation between NAFLD presence and clinicopathological features was summarized in Table 2.

### Survival Analyses

**Figure 2:**
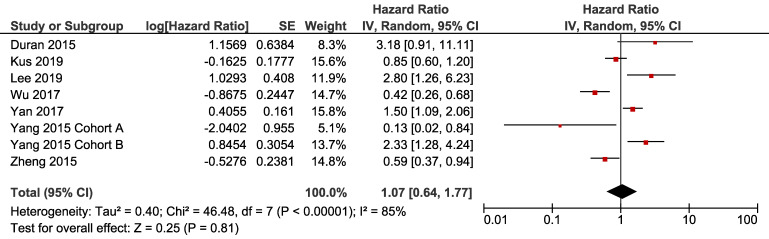
** Forest plot of HR for DFS.** Square indicate point estimate of each study. Size of square indicates relative contribution of each study. Solid horizontal line represents 95% CI of each study. Diamond indicates pooled studies.

For disease free survival, all the eight cohorts from seven studies were included for meta-analysis. Three cohorts showed that patients with NAFLD had poorer DFS compared to the non-NAFLD group, while the other three cohorts reached the contradictory conclusion. Pooled result (heterogeneity analysis: I-square = 85%, Cochrane's Q *p* < 0.001) revealed that NAFLD had no significant impact on DFS [HR 1.07, 95% CI = 0.64-1.77, *p* = 0.81] (Fig. 2).

**Figure 3:**
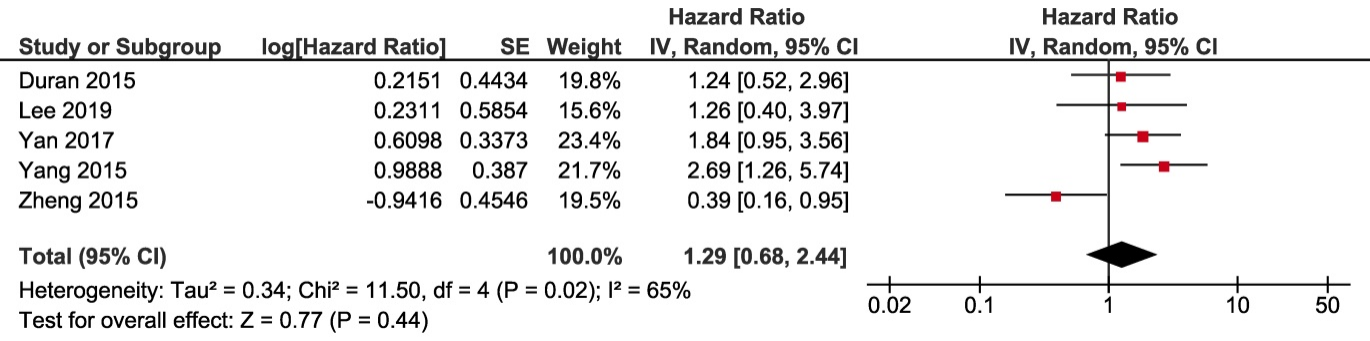
** Forest plot of HR for OS.** Square indicate point estimate of each study. Size of square indicates relative contribution of each study. Solid horizontal line represents 95% CI of each study. Diamond indicates pooled HR value.

For overall survival, five cohorts had available data, with significant heterogeneity (I-square = 65%, Cochrane's Q *p* = 0.02). Pooled result revealed that NAFLD had no significant impact on OS of breast cancer (HR 1.29, 95% CI = 0.68-2.44, *p* = 0.44) (Fig. 3).

### Subgroup Analysis

**Figure 4:**
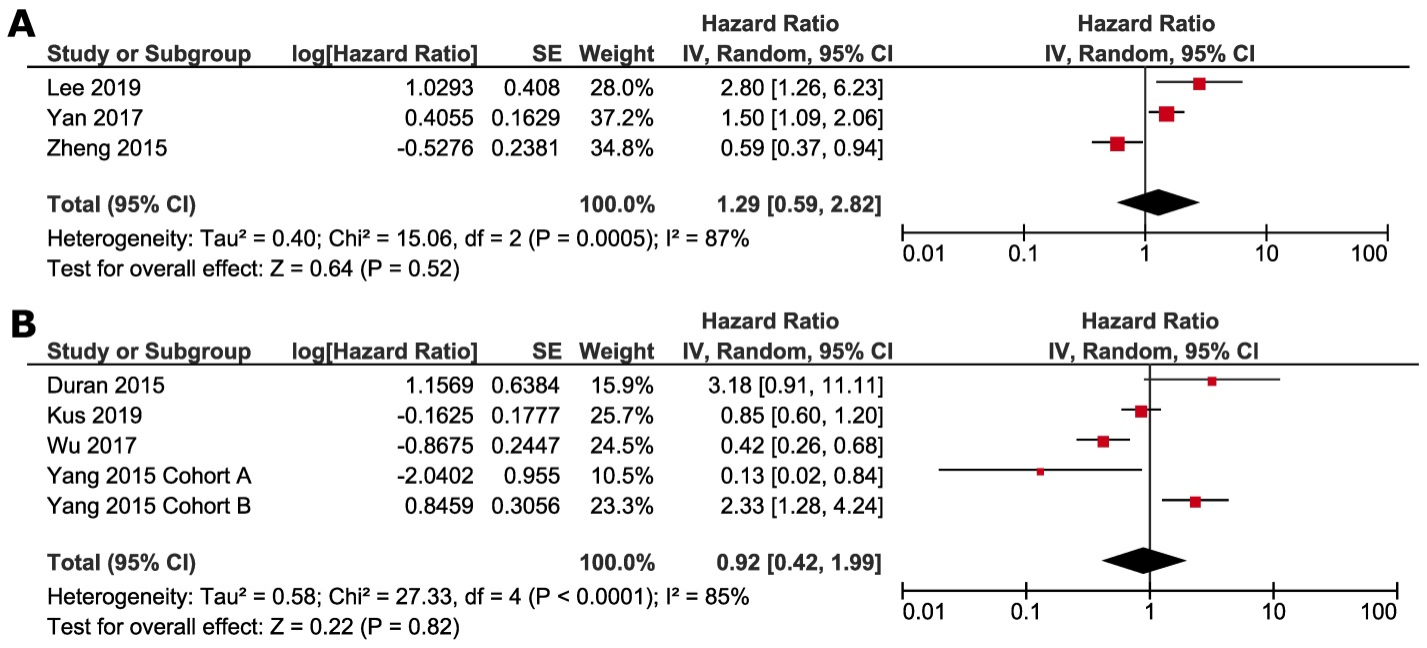
** Subgroup analysis of DFS according to whether NAFLD was associated with ET: A. ET-associated NAFLD; B. non-ET associated NAFLD.** Square indicate point estimate of each study. Size of square indicates relative contribution of each study. Solid horizontal line represents 95% CI of each study. Diamond indicates pooled HR value.

**Figure 5:**
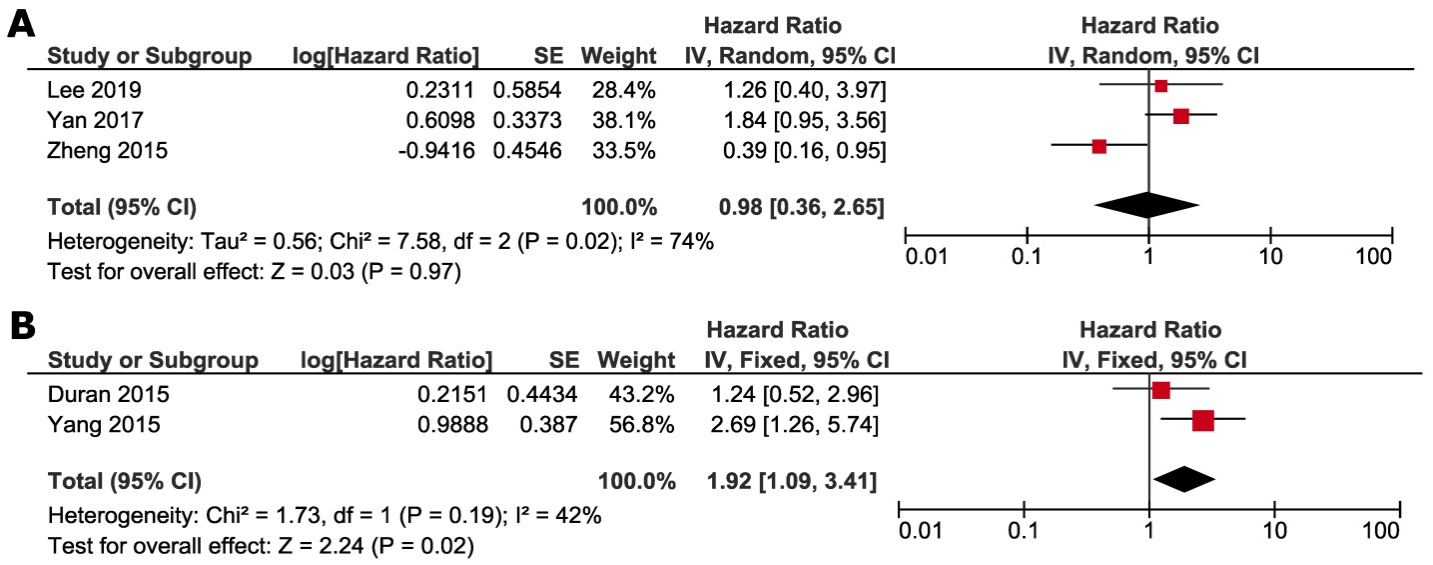
** Subgroup analysis of OS according to whether NAFLD was associated with SERM: A. ET-associated NAFLD; B. non-ET associated NAFLD.** Square indicate point estimate of each study. Size of square indicates relative contribution of each study. Solid horizontal line represents 95% CI of each study. Diamond indicates pooled HR value.

Subgroup analyses were conducted according to whether NAFLD was induced by ET or not. Studies by Zheng *et al.*, Lee *et al.* and Yan *et al.* focused on the impact of ET-associated NAFLD, and other studies selected the patients not specified to previous ET. ET-associated NAFLD had no significant impact on DFS (HR 1.29, 95% CI=0.59 - 2.82, *p* = 0.52) (Fig. 4A) and OS (HR 0.98, 95% CI =0.36-2.65, *p* = 0.97) (Fig. 5A). For subgroup of non-ET-associated NAFLD, the pooled result also had no significant impact on DFS (HR 0.92, 95% CI=0.42-1.99, p=0.82) (Fig. 4B). However, OS data proved that patients with non-ET-associated NAFLD had increased death risk compared to the patients without NAFLD (HR 1.92, 95% CI = 1.09-3.41, *p* = 0.02) (Fig. 5B).

### Publication Bias

Publication bias was investigated by funnel plots for DFS (Supplementary Fig. 1) and OS (Supplementary Fig. 2). Begg's Tests were conducted and revealed no significant publication bias (DFS: *p* = 0.711 and OS: *p* = 0.462).

## Discussion

Nowadays, NAFLD has become a common disorder, especially the wide use of ET could be an independent risk factor of NAFLD for breast cancer patients^18^. Study by Pan *et al.* suggested that tamoxifen could increase the risk of newly developed NAFLD or worsening pre-existed conditions, even compromised treatment efficacy^9^. The impact of NAFLD on breast cancer survival remained unclear and arose special attention recently^16^.

As the first meta-analysis regarding the prognostic value of NAFLD on breast cancer survival, we enrolled seven studies with eight cohorts and 3684 patients, the results illustrated that NAFLD presence correlated with advanced age (> 50 years), higher BMI (> 25 kg/m^2^), lymph node involvement and hormone-receptor positivity. Despite the including studies drew contradictory conclusions regrading NAFLD prognostic value, the pooled result proved NAFLD had no impact on DFS or OS for breast cancer patients. Moreover, subgroup analysis on whether patients received ET was also conducted. In subgroup with ET related NAFLD, the onset of NAFLD after the ET administration showed no impact on survival in terms of BCSS and OS, indicating that NAFLD as a common adverse effect of endocrine therapy, may have little influence on breast cancer prognosis. While, non-ET-associated NAFLD had a strong correlation with shortened OS, indicating its correlation with increasing mortality.

NAFLD is a chronic liver disease ranging from simple steatosis to non-alcoholic steatohepatitis^19^. It is considered to incorporate with many risk factors like obesity, insulin resistance or even type 2 diabetes^20^. The mechanism of NAFLD was not fully understood, but a two-hit theory had been proposed: the first hit assumed to be lipid accumulation in the liver, and the second hit was oxidative stress^21^. As liver was a prime target of excessive lipid storage in obesity, the occurrence of NAFLD needed long-term progression, such as lipid accumulation, adipose-derived inflammation, and those risk factors may result in strong correlation between NAFLD and advanced age (age > 50-years) (*p* < 0.001) or obesity (BMI > 25 kg/m^2^) (*p* < 0.001)^22^. Furthermore, tamoxifen was also associated with lipid metabolism disorder, as it increased serum triglycerides and inhibit fatty acid beta-oxidation^23^-^25^. This was consistent with the notion that tamoxifen played a key role in the first hit: the deposition of fat in the liver. Increased cytokine activity, oxidative stress, and mitochondrial dysfunction were implicated in the second hit^10^. This was in line with our results that advanced age, obesity, hormone receptor-positive all contributed to the NAFLD occurrence.

In our meta-analysis, the pooled result proved that NAFLD had no impact on breast cancer DFS and OS. This conclusion was consistent with study by Duran *et al*. 16 . And study by Wu *et al.* even reported fatty liver decreased the risk of liver metastasis in patient with breast cancer^12^. Although NAFLD was considered to be part of metabolic syndromes and increase cancer risk^26^, its negative impact on survival could be potentially contradicted by its protective effect from liver metastases. Another reasonable explanation lies in the wide use of ET and corresponding increase of ET-associated NAFLD. NAFLD was regarded as a common side effect of ET, and it could be speculated that ET-induced NAFLD may have different pathophysiological mechanism and prognostic value compared with general NAFLD. And this was also supported by our subgroup analysis that ET-associated NAFLD had no significant impact on breast cancer prognosis in terms of both DFS and OS.

Long-term administration of ET, including Tamoxifen and Toremifene, could cause a wide spectrum of adverse effects, including pulmonary embolism, deep vein thrombosis, stroke, NAFLD, and climacteric symptoms 27,28. Regarding NAFLD, studies by Zheng *et al.* and Yan *et al.* focused on ET-associated NAFLD and drew contradictory conclusions. Studies by Zheng *et al.* indicated that ET-associated NAFLD could be a protective factor both on DFS and OS^13^. In subgroup analyses of the present meta-analysis, ET-associated group showed no significant difference of OS between NAFLD and non-NAFLD patients. Since NAFLD as a frequent ET-related adverse event usually compromised the patients' compliance for long-term endocrine therapy, the present study supported the notion that NAFLD during ET therapy may not be a big concern that lead to discontinuation of endocrine therapy. Clinicians could adopt individual and personalized strategy on whether to cease endocrine therapy due to ET-associated NAFLD.

The molecular mechanisms underlying NAFLD and cancer survival were still controversial. One of the possible mechanisms may involve the insulin and insulin-like growth factor (IGF) axis and chronic inflammation. Insulin and IGF-1 receptors(IGF-1R) were important activators of the Akt and mitogen-activated protein kinase(MAPK) signaling networks in neoplastic tissue^29^,^30^, and these pathways were demonstrated to mediate antiestrogen resistance via cross talk with ER signaling^31^. In Vitro study proved that overexpression of IGF-1R in MCF7 cells increased receptor tyrosine kinase activity in response to IGF-1 ligand stimulation and enhanced antiestrogen resistance^31^. Since IGF-1 level were reduced in NAFLD patients 32, it could partially explain why ET-associated NAFLD had no significant impact on breast cancer outcomes.

On the other hand, non-ET-associated NAFLD group showed a shorter overall survival than non-NAFLD group. It is concordant with the finding that metabolic syndrome including diabetes, NAFLD and so on could increase breast cancer risk 26. This result implies that pre-existing NAFLD before administration of endocrine therapy is of prognostic value associated with poor prognosis, indicating intensive control for metabolism syndromes with diet and lifestyle change, even medication, could benefit patient's survival.

This meta-analysis was restricted by the limited number of eligible studies, which partially and inevitably induced heterogeneity and bias to our results. Besides, lack of individual data also limited the analysis of clinicopathological features in NAFLD and non-NAFLD patients and restricted further subgroup analysis, sensitivity analysis and publication bias evaluation. All the studies included in the present meta-analysis only have prognostic information on overall study population and did not provide survival data on individual molecular subtypes, such as triple-negative and HER2-rich subgroups. So meta-analyses on these two subtypes and direct comparison between ER+ and ER- breast cancer were unable to perform based on the available data. Future large-scale studies are warranted to further elucidate the correlation between NAFLD and breast cancer prognosis.

## Conclusion

Our study suggested that, in general, presence of NAFLD had no impact on DFS or OS of breast cancer patients. Similarly, the ET-associated NAFLD has no significant survival difference compared to non-NAFLD patients. However, patients with non-ET-associated NAFLD had increased death risk in terms of shortened OS. As for non-ET-associated NAFLD patient, clinicians should be more vigilant and encouraged to perform active treatment to control NAFLD and metabolic syndrome.

## Supplementary Material

Supplementary figures and information.

## Figures and Tables

**Table 1:** Characteristics of studies enrolled in meta-analysis

Study	Country	Design	N	Median follow-up (month)	TNM Stage	NAFLD Assessment	Clinical Endpoints	Hormone Receptor	Relationship Between SERM-treatment and NAFLD
Duran (2015)	Turkey	RC	107	NA	IV	Contrast-enhanced/ Non-Contrast CT	OS	NS	Non-SERM-associated NAFLD
Kus (2019)	Turkey	RC	424	80.4(7.2-156)	IV	USG	DFS	NS	NS
Lee (2019)	Korea	RC	440	100.8(9.6-138)	I-III	USG	DFS, OS	ER/PR+	AI-associated NAFLD
Wu (2017)	China	RC	1230	30.7±24.9 32.4±26.3	I-III	USG	Liver-MFS	NS	Non-SERM-associated NAFLD
Yan (2017)	China	RC	646	64(7-91)	I-III	USG	DFS, OS	ER/PR+	SERM-associated NAFLD
Yang (2015) Cohort A^*^	Korea	RC	28	NA	II/III	USG/ Non-Contrast CT/ Contrast CT	DFS, OS	NS	Non-SERM-associated NAFLD
Yang (2015) Cohort B^*^	Korea	RC	24	NA	II/III	USG/ Non-Contrast CT/ Contrast CT	DFS, OS	NS	Non-SERM-associated NAFLD
Zheng (2015)	China	RC	785	76(14-122)	I-III	USG	DFS, OS	ER/PR+	SERM-associated NAFLD

RC, retrospective cohort; NS, not specified; CT, computed tomography; OS, overall survival; DFS, disease free survival; SERM, selective estrogen receptor modular; AI, aromatase inhibitor; USG, ultrasonography; MRI, Magnetic resonance imaging; MFS, metastasis free survival. *Study by Yang et al. included two cohorts

**Table 2:** Correlation Between NAFLD Presence and Clinicopathological Features

	NAFLD(%)		Non/mild-NAFLD(%)		P value
Age (years)					
<50	354	(24.7%)	1081	(75.3%)	<0.001^*^
>50	397	(32.4%)	829	(67.6)	
BMI (kg/m^2^)					
<25	566	(25.4%)	1665	(74.6%)	<0.001^*^
>25	405	(46.7%)	463	(53.3%)	
Tumor Size (mm)					
<20	293	(27.1%)	790	(72.9%)	0.472
20-50	378	(28.8%)	935	(71.2%)	
>50	80	(30.3%)	184	(69.7%)	
LN Metastasis					
Yes	540	(40.9%)	780	(59.1%)	0.003^*^
No	281	(36.6%)	486	(63.4%)	
Menopause					
Pre	240	(35.3%)	439	(64.7%)	0.002^*^
Post	401	(43.0%)	531	(57.0%)	
ER/PR Status					
ER/PR +	630	(34.9%)	1173	(65.1%)	<0.001^*^
ER&PR -	121	(14.1%)	737	(85.9%)	
HER2					
Positive	331	(30.0%)	773	(70.0%)	0.304
Negative	371	(28.0%)	954	(72.0%)	
Radiotherapy					
Yes	215	(27.5%)	566	(72.5%)	0.608
No	536	(28.5%)	1344	(71.5%)	

*indicates statistical significance.

## Abbreviations

NAFLD: Non-alcoholic fatty liver disease
ET: endocrine therapy
DFS: disease free survival
OS: overall survival
HR: hazard ratio
CI: confidence interval
NASH: nonalcoholic steatohepatitis
SERM: selective-estrogen receptor modulator
AI: aromatase inhibitor
CENTRAL: Central Register of Controlled Trials
