# Supplementary material for: The impact of pre-existed and SERM-induced non-alcoholic fatty liver disease on breast cancer survival: a meta-analysis
Source: J Cancer. 2020 May 18;11(15):4597–604. doi: 10.7150/jca.44872 (PMC7255364; doi:10.7150/jca.44872)
Supplement: Supplementary file 1 — Supplementary figures and information. [file jcav11p4597s1.pdf]

Supplementary material

Supplementary Figure 1 Funnel plot for potential publication bias of DFS

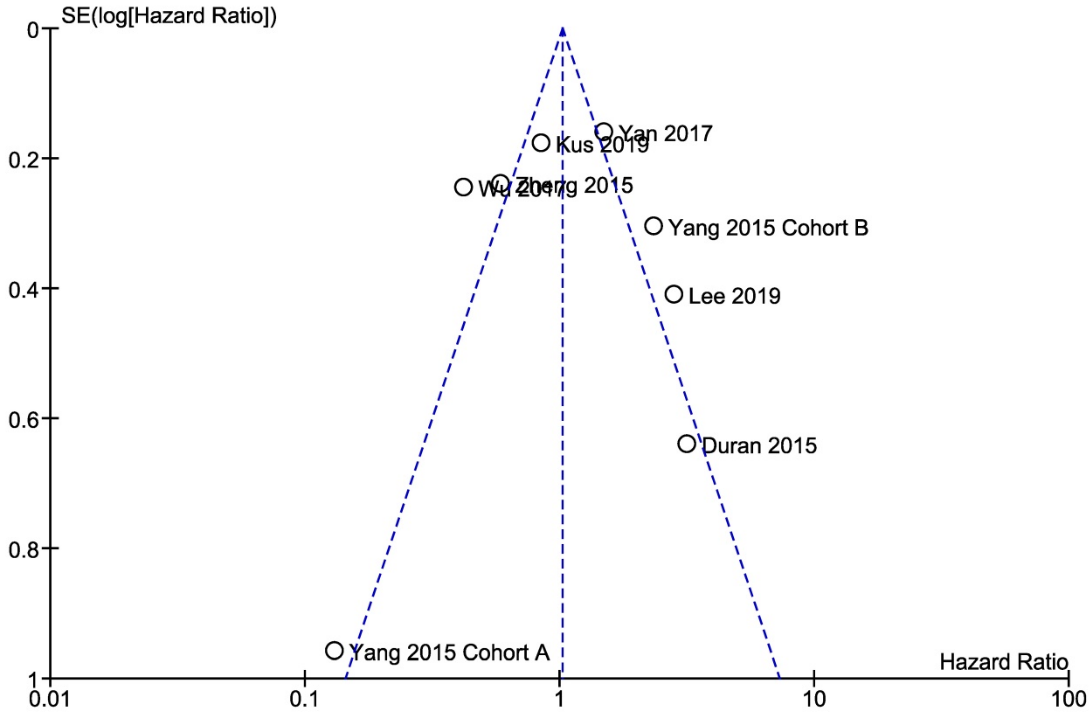

Supplementary Figure 2 Funnel plot for potential publication bias of OS

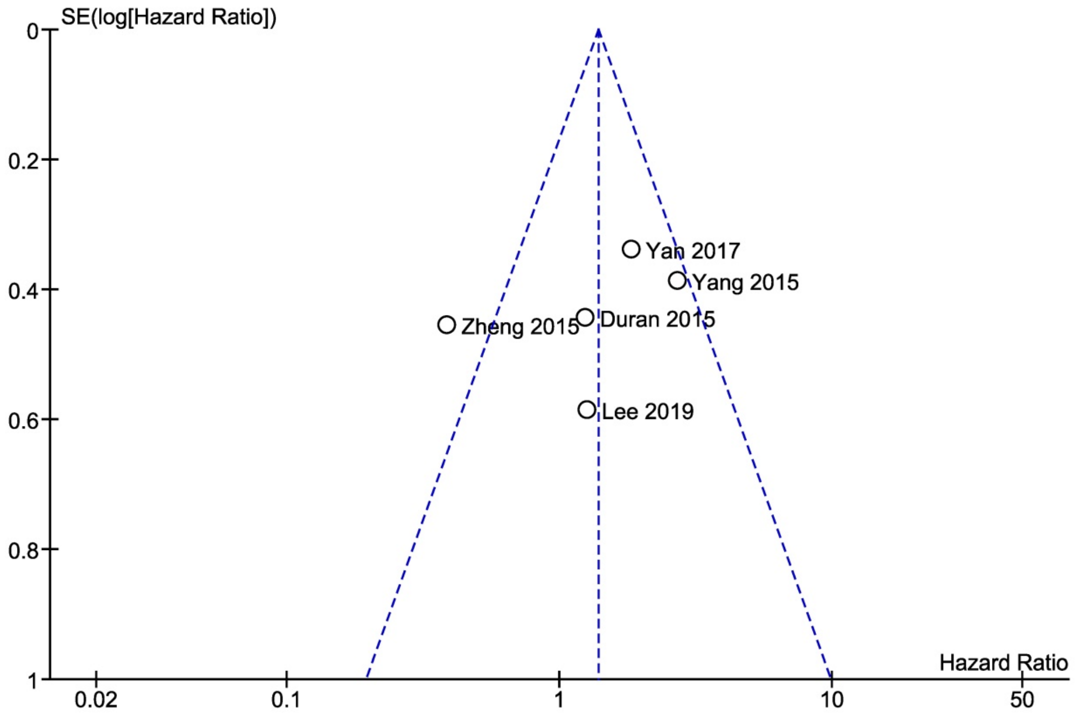

- ## Search Strategy for Pubmed Database

  Search ((((((((((((((((((((((((((((((((((((((("Breast Neoplasms"[Mesh]) OR Tumors, Breast) OR Breast Tumors) OR Breast Tumor) OR Tumor, Breast) OR Neoplasms, Breast) OR Breast Neoplasm) OR Neoplasm, Breast) OR Breast Cancer) OR Cancer, Breast) OR Cancer of the Breast) OR Mammary Cancer) OR Cancer, Mammary) OR Cancers, Mammary) OR Mammary Cancers) OR Malignant Neoplasm of Breast) OR Breast Malignant Neoplasm) OR Breast Malignant Neoplasms) OR Malignant Tumor of Breast) OR Breast Malignant Tumor) OR Breast Malignant Tumors) OR Cancer of Breast) OR Breast Carcinoma) OR Breast Carcinomas) OR Carcinoma, Breast) OR Carcinomas, Breast) OR Mammary Carcinoma, Human) OR Carcinoma, Human Mammary) OR Carcinomas, Human Mammary) OR Human Mammary Carcinomas) OR Mammary Carcinomas, Human) OR Human Mammary Carcinoma) OR Mammary Neoplasms, Human) OR Human Mammary Neoplasm) OR Human Mammary Neoplasms) OR Neoplasm, Human Mammary) OR Neoplasms, Human Mammary) OR Mammary Neoplasm, Human)) AND (((((((((((((("Non-alcoholic Fatty Liver Disease"[Mesh]) OR Non alcoholic Fatty Liver Disease) OR NAFLD) OR Nonalcoholic Fatty Liver Disease) OR Fatty Liver, Nonalcoholic) OR Fatty Livers, Nonalcoholic) OR Liver, Nonalcoholic Fatty) OR Livers, Nonalcoholic Fatty) OR Nonalcoholic Fatty Liver) OR Nonalcoholic Fatty Livers) OR Nonalcoholic Steatohepatitis) OR Nonalcoholic Steatohepatitides) OR Steatohepatitides, Nonalcoholic) OR Steatohepatitis, Nonalcoholic)
